# Dysport and Botox at a Ratio of 2.5:1 Units in Cervical Dystonia: A Double-Blind, Randomized Study

**DOI:** 10.1002/mds.26085

**Published:** 2014-12-05

**Authors:** Ji Young Yun, Jae Woo Kim, Hee-Tae Kim, Sun Ju Chung, Jong-Min Kim, Jin Whan Cho, Jee-Young Lee, Ha Neul Lee, Sooyeoun You, Eungseok Oh, Heejeong Jeong, Young Eun Kim, Han-Joon Kim, Won Yong Lee, Beom S Jeon

**Affiliations:** 1 Department of Neurology, Ewha Womans University School of Medicine and Ewha Medical Research InstituteSeoul, Republic of Korea; 2 Department of Neurology, Parkinson‘s Disease Centre, Dong-A University HospitalPusan, Republic of Korea; 3 Department of Neurology, College of Medicine, Hanyang UniversitySeoul, Republic of Korea; 4 Department of Neurology, Asan Medical Center, University of Ulsan College of MedicineSeoul, Republic of Korea; 5 Department of Neurology, Seoul National University Bundang HospitalSeongnam, Republic of Korea; 6 Department of Neurology, Samsung Medical Center, Sungkyunkwan University School of MedicineSeoul, Republic of Korea; 7 Department of Neurology. Seoul National University-Seoul Metropolitan Government Boramae Medical CenterSeoul, Republic of Korea; 8 Department of Neurology, Dongsan Medical Center, Keimyung UniversityDaegu, Republic of Korea; 9 Department of Neurology, Chungnam National University Hospital, Chungnam National University School of MedicineDaejeon, Republic of Korea; 10 Department of Neurology, Gyeongsang National University School of MedicineJinju, Republic of Korea; 11 Department of Neurology, Hallym University Sacred Heart Hospital, Hallym University College of MedicineAnyang, Republic of Korea; 12 Department of Neurology and Movement Disorder Center, Parkinson study group, and Neuroscience Research Institute, College of Medicine, Seoul National UniversitySeoul, Republic of Korea

**Keywords:** cervical dystonia, torticollis, botulinum toxin, motor control, movement disorders

## Abstract

We aimed to compare Dysport (abobotulinumtoxinA, Ipsen Biopharm, Slough, UK) and Botox (onabotulinumtoxinA, Allergan, Irvine, CA, USA) at a 2.5:1 ratio in the treatment of cervical dystonia (CD). A Dysport/Botox ratio of lower than 3:1 was suggested as a more appropriate conversion ratio, considering its higher efficacy and more frequent incidence of adverse effects not only in the treatment of CD but also in other focal movement disorders. A randomized, double-blind, multicenter, non-inferiority, two-period crossover study was done in CD, with a duration of at least 18 months. Patients were randomly assigned to treatment for the first period with Dysport or Botox, and they were followed up for 16 weeks after the injection. After a 4-week washout period, they were switched to the other formulation and then followed up for 16 weeks. The primary outcome was the changes in the Tsui scale between the baseline value and that at 1 month after each injection. A total of 103 patients were enrolled, and 94 completed the study. Mean changes in the Tsui scale between baseline and 4 weeks after each injection tended to favor Botox; however, this was not statistically significant (4.0 ± 3.9 points for the Dysport treatment vs. 4.8 ± 4.1 points for Botox; 95% confidence interval, −0.1-1.7; *P* = 0.091). The mean change of the Toronto western spasmodic torticollis rating scale score, the proportion of improvement in clinical global impression and patient global impression, and the incidences of adverse events were not significantly different between the two treatments. With regard to safety and efficacy, Dysport was not inferior to Botox in patients with CD at a conversion factor of 2.5:1. [http//clinicaltrial.gov: NCT00950664] © The Authors. Movement Disorders published by Wiley Periodicals, Inc. on behalf of International Parkinson and Movement Disorder Society.

Cervical dystonia (CD) is characterized by involuntary contraction of the neck muscles, frequently leading to neck pain, disability, abnormal posture, and social withdrawal.^1^ At present, botulinum toxin type A (BoNT-A) is recommended as the first-line treatment option for CD.^2^

Several commercially available formulations of BoNT-A are available for CD treatment: Dysport (abobotulinumtoxinA, Ipsen Biopharm, Slough, UK), Botox (onabotulinumtoxinA, Allergan, Irvine, CA, USA), and Xeomin (incobotulinumtoxinA, Merz Pharmaceuticals GmBH, Frankfurt, Germany).

International units are used to express the potency of these formulations. However, the units for each product are not equivalent, and debate has arisen concerning the optimal unit conversion ratio between Dysport and Botox.^3^ The Dysport/Botox conversion ratio has ranged from 1.7:1 to 5:1 in the treatment of CD.^4^–^6^

Two double-blind, randomized clinical trials have been performed to investigate treatment equivalency and Dysport/Botox conversion ratios.^7^,^8^ A ratio of 3:1 was suggested.^7^ However, Ranoux et al.^8^ proposed that ratios lower than 3:1, if producing equivalent efficacies, may be preferred because of the frequent adverse effects (AE) at the ratios used in their studies.^8^ Other authors have also suggested that the optimal ratios may be lower than 3:1,^3^,^9^ with one study suggesting a range of 2 to 2.5:1.^10^

Botox is usually supplied in 100-unit vials and Dysport in 500-unit vials, and the average recommended dose for patients with CD is 200 units of Botox (2 vials) or 500 units of Dysport (1 vial).^11^ On that basis, the conversion rate is 2.5:1. Assuming a bioequivalence ratio of 2.5:1 units in the treatment of CD, we undertook a double-blind, randomized, multi-center, crossover study to determine the non-inferiority of Dysport in clinical efficacy and safety, in comparison with Botox, when treating at a 2.5:1 conversion ratio.

## Methods

### Patients

The CD patients in this study were recruited in movement disorder clinics at participating centers from August 2009 to March 2010. Patients with CD were eligible for enrollment in this study if they were older than 20 years and had CD duration of 18 or more months. The oral CD medications dosages were kept stable throughout the study.

Patients were excluded if they had a history of clinically significant dysphagia due to BoNT-A injection requiring a Levin tube; a diagnosis of myasthenia gravis or other disease of the neuromuscular junction; a diagnosis of cervical contracture; or if they were currently pregnant or breastfeeding. Patients were also excluded if they underwent previous myotomy to cervical muscles, phenol injections, or denervation surgery involving the cervical region. Patients who required a BoNT-A dose of greater than 200 units as Botox or greater than 500 units as Dysport were excluded. Because nearly all Korean CD patients receive Dysport less than 500 units or Botox less than 200 units, no patient was excluded in the screening because of these criteria. Patient who had participated in other clinical trials in the 4 months preceding this study's enrollment period were also not permitted to participate. Patients treated previously with BoNT-A were allowed to participate if at least 16 weeks had elapsed since the last injection.

All participating centers obtained the approval of their institutional review boards and conformed to the principles of the Declaration of Helsinki. All patients signed informed consents before participation in this study. The study was registered at http://www.ClinicalTrials.gov (identifier: NCT00950664).

### Randomization and Intervention

Initially, all patients were randomly assigned to either the Dysport or Botox treatment group by using a predetermined randomization table. To ensure blindness, the following measures were undertaken:

The randomization code was generated by the Medical Research Collaborating Center at Seoul National University Hospital before study initiation. Randomization of the injection order was carried out by using a permuted-block design with a block size of 4 and 6. Assignments were not stratified by enrolling centers.

The randomization table was transferred by the Medical Research Collaborating Center at Seoul National University Hospital directly to the pharmacy of each clinical trial center. All patients and investigators except for the pharmacists were blinded from assignment until all data were ready for analysis after study completion. The pharmacists were not involved in any patient's treatment or assessment.

Both formulations were diluted in a room separated from all investigators and patients, by the pharmacists. To ensure blindness, 2 mL normal saline was used to dilute one vial of 500-unit Dysport and two vials of 100-unit Botox at a Dysport/Botox unit ratio of 2.5:1. To ensure the same injection volumes for the two crossover injections, those dilutions were then drawn into three 1-mL syringes containing 0.6 mL, 0.6 mL, and 0.4 mL.

The syringes were delivered to the injectors, who were blinded to the contents of the syringes. During the study, 0.1 to 0.2 mL was administered at each injection site. Treatment-related training was performed twice before initiating the study, and once during the study.

### Study Design

**Figure 1:**
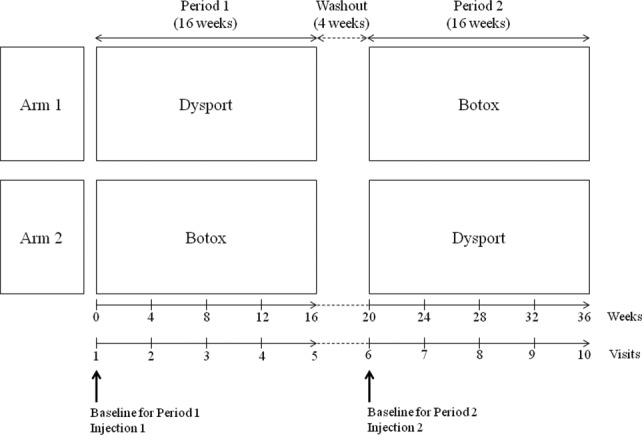
Study design.

Patients received either Dysport or Botox injection on assignment during the first study period. The subjects were followed up once per month for 16 weeks in the first study period. After a 4-week washout period, those who received Dysport were reassigned Botox, and vice versa. To avoid carryover effects in this crossover design, we planned a 16-week treatment period followed by a 4-week washout period, even though the benefit of an injection for CD lasts generally approximately 14 weeks^11^ (Fig. 1).

Doses of BoNT-A received by patients were determined a priori and fixed by the investigators at a dose of 2.5:1 for Dysport and Botox, respectively. The volume, sites, and number of injections into each muscle were the same between the two regimens. For each patient, injectors were the same in the crossover. Subjects were also followed up monthly for 16 weeks in the second period. The results from both periods were merged and compared according to the two different formulations.

### Clinical Assessments

In each treatment group, monthly changes from baseline at follow-up weeks 4, 8, 12, and 16 were assessed by using the Tsui scale^12^ and the Toronto western spasmodic torticollis rating scale (TWSTRS).^13^

At each visit, including baseline, the investigators obtained clinical global impression (CGI) and patients assessed their global impression (PGI) for each treatment. A patient's preference for treatments types was asked at trial completion. The preference was assessed on the basis of response to the question: “Which regimen do you prefer?” The choices of answers were: “I prefer the first one”, “I prefer the second one” or “I do not prefer one treatment over the other,” in addition to reasons for their preference.

### Efficacy Assessments

The primary efficacy outcome was the change in the Tsui scale between the baseline value and that at 1 month after each injection (peak effect).^14^,^15^ Secondary outcomes included analysis of changes in the TWSTRS between baseline and 1 month after each injection and the proportions of patients with CGI of illness (CGI-I) and PGI of improvement (PGI-I). We also recorded all changes in Tsui and TWSTRS scales and the proportion of CGI-I and PGI-I at each visit. Additionally, we analyzed improvements in the TWSTRS subscale for severity, disability, and pain at each visit and the proportions of patient with a preference for Dysport or Botox. Safety was assessed by the incidence of AE reported throughout the study.

### Sample Size and Statistical Analysis

The required sample size calculation was based on the previous studies investigating a mean change in Tsui score between baseline and 4 weeks after first injection.^7^,^8^ Assuming that a between-treatment difference in Tsui score was 1.5 points based on the study by Ranoux et al,^8^ and using one-sided test with a significance level of 0.05 and 80% power, the target sample size of 79 patients was estimated. The required patient number also assumed an estimated 25% attrition rate.

The study was designed to test the hypothesis that the change in Tsui score from baseline achieved with Dysport is non-inferior to that of Botox in a 2.5:1 ratio at the end of 4 weeks of treatment. For the primary outcome, non-inferiority of Dysport/Botox at a ratio of 2.5:1 was concluded if the upper limit of the one-sided 95% confidence interval (CI) for the difference between treatment (Dysport minus Botox) in change from baseline as Tsui score was 1.5 points or less. For scale variables, including changes in Tsui scale and total score and subscales in TWSTRS from baseline, the data were analyzed by paired *t* tests between baseline and 4 weeks after each injection.

McNemar chi-square tests were used to evaluate categorical values, such as differences in CGI-I or PGI-I proportions and in the incidence of overall AEs between the treatment groups. For assessment of patients' preference, descriptive statistics were used.

Two study populations were defined. The modified intent-to-treat population (mITT) consisted of all randomized patients who completed the Tsui scale at baseline and at least once after 4 weeks of treatment in each crossover period. The per-protocol population (PP) consisted of patients in the mITT who had no protocol violations and who completed the Tsui scale at all visits.

In our crossover design, to test for presence of a carryover effect, changes in Tsui score obtained immediately before each intervention were compared, and the Mann-Whitney *U* test was used to assess differences between the patients switching from Dysport to Botox (arm 1) and those changing from Botox to Dysport (arm 2).

All statistical analyses were performed by using SPSS 21.0. A *P*-value < 0.05 was considered statistically significant.

## Results

### Patient Disposition and Characteristics

**Figure 2:**
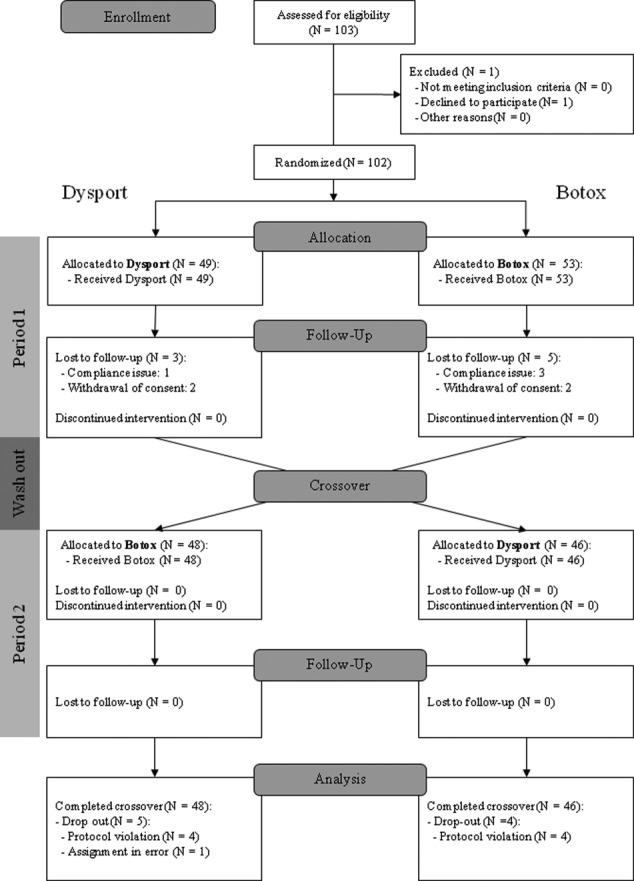
CONSORT 2012 flow diagram of patients.

Of the 103 patients originally enrolled from seven centers, one declined to participate before initiation of randomization. Thus, 102 subjects were randomized, with 49 and 53 allocated to the arm 1 and arm 2 groups, respectively. In arm 1, patients received Dysport during the first injection period (period 1); two of those patients withdrew consent after the intervention, and one patient discontinued the study because of noncompliance. During the second injection period (period 2) of arm 1, the remaining 46 patients received Botox, and all completed the intervention. In period 1 of arm 2, all participants received Botox; two of those patients withdrew consent in subsequent visits, and three subjects discontinued the study because of poor compliance. After the washout period, the remaining 48 patients received Dysport and completed the study (Fig. 2). The two groups were similar at baseline with respect to demographic and clinical characteristics (Table1).

**Table 1:** Baseline characteristics and disease characteristics in the modified intent-to-treat population.

Parameter	Both Arms (N = 94)	Arm 1 (N = 46)	Arm 2 (N = 48)	*P* Value
		Dysport → Botox	Botox → Dysport	Arm 1 vs. Arm 2
Age at visit 1 (y)	53.30 ± 10.76	53.24 ± 11.44	53.35 ± 10.18	0.433
Sex (M:F)	37 : 57	20 : 26	17 : 31	0.527
Time since onset of cervical dystonia (y)	10.46 ± 8.62	11.36 ± 9.21	9.57 ± 8.00	0.285
Weight at visit 1 (kg)	60.15 ± 8.27	60.03 ± 7.33	60.25 ± 9.14	0.181
Height at visit 1 (cm)	162.32 ± 7.39	162.66 ± 7.08	162.00 ± 7.73	0.596
Patients with previous BoNT-A treatment before entry (%)	40 (42.6)	17 (37.0)	23 (47.9)	0.304
Time since recent BoNT-A treatment before entry (months)	19.59 ± 27.96 (Range: 5-150)	12.62 ± 9.56 (Range: 5-40)	24.37 ± 35.01 (Range: 5-150)	0.500
Tsui score	11.12 ± 4.37	11.22 ± 4.45	11.02 ± 4.34	0.285
TWSTRS	34.90 ± 13.33	35.41 ± 14.83	34.39 ± 11.85	0.081
TWSTRS severity subscale	16.73 ± 5.72	17.46 ± 6.05	16.04 ± 5.35	0.180
TWSTRS disability subscale	11.41 ± 5.37	11.59 ± 5.90	11.25 ± 4.88	0.350
TWSTRS pain subscale	6.74 ± 4.76	6.37 ± 5.05	7.10 ± 4.48	0.096
Number of patients (%) scoring 1 or 2 or 3 in CGI (CGI-I)	18 (19.1)	10 (21.7)	8 (16.7)	0.605

				*P* value
Mean baseline score in each treatment		Dysport (N = 94)	Botox (N = 94)	Dysport vs. Botox
Tsui score		10.51 ± 4.66	10.73 ± 4.54	0.551
TWSTRS		33.01 ± 13.90	32.49 ± 12.65	0.613
TWSTRS severity subscore		16.63 ± 6.21	15.88 ± 5.85	0.095
TWSTRS disability subscore		10.76 ± 5.52	10.62 ± 4.93	0.750
TWSTRS pain subscore		5.61 ± 4.84	6.12 ± 4.63	0.247
Number of patients (%) scoring 1 or 2 or 3 in CGI (CGI-I)^a^		25 (26.6)	24 (25.5)	1.000

BoNT-A, Botulinum toxin type A; TWSTRS, Toronto western spasmodic torticollis rating scale; CGI, clinical global impression; CGI-I, clinical global impression of illness.

a CGI-I is the proportions of patients with CGI of illness of ‘1 = normal/not at all ill'or ‘2 = borderline mildly ill,' or ‘3 = mildly ill.'

A total of 94 patients completed both interventions and participated in all study evaluations. After trial, however, one patient's disease duration was corrected to 8 months, thus not meeting enrollment indication. Moreover, injection sites of eight patients were not kept the same between the two phases, thus violating the study protocol. We included all of the patients who completed two treatments in the study plan; these nine patients were included in the mITT. Eight dropouts in period 1 were excluded from analysis because they did not complete both treatments (Fig. 2). Patients received a mean dose of 361.04 ± 57.91 (range, 200-400) units of Dysport and 144.41 ± 23.16 of Botox (80-160) in each period.

### Outcome Measures

A tendency was seen to favor Botox in the mean changes in the Tsui scale between baseline and 4 weeks after each injection; however, it was not statistically significant (4.0 ± 3.9 points for Dysport vs. 4.8 ± 4.1 points for Botox; 95% confidence interval, −0.1 to 1.7; *P* = 0.091). In addition, no significant difference was found between the two formulation groups in mean reduction in TWSTRS (95% CI for difference, −3.4 to 1.5, *P* = 0.429) from the baseline to 4 weeks after each injection (Table2). At other follow-up visits, similar results were obtained for changes in the Tsui scale and TWSTRS from baseline (Supplemental Data Table e-1).

**Table 2:** Clinical outcomes after 4 weeks from baseline in the modified intent-to-treat population

Scale	Dysport (n = 94)	Botox (n = 94)	Between-treatment difference in end-point	*P* value
Mean changes of Tsui from baseline	−3.98 ± 3.89	−4.77 ± 4.10	0.78 [-0.13 to 1.70]	0.091
Mean changes of Total TWSTRS from baseline	−9.76 ± 10.25	−8.78 ± 10.11	−0.97 [-3.39 to 1.45]	0.429
Mean changes of TWSTRS severity subscore	−5.55 ± 4.99	−5.26 ± 4.79	−0.30 [-1.46 to 0.86]	0.611
Mean changes of TWSTRS disability subscore	−2.76 ± 3.64	−2.46 ± 3.60	−0.30 [-1.23 to 0.64]	0.529
Mean changes of TWSTRS pain subscore	−1.45 ± 4.05	−1.19 ± 4.16	−0.25 [-1.28 to 0.77]	0.623
Number of patients (%) scoring 1 or 2 or 3 on CGI scale (CGI-I)^a^	54/94 (57.4 %)	57/94 (60.6 %)		0.648
Number of patients (%) scoring 1 or 2 or 3 on PGI scale (PGI-I)^a^	75/94 (79.8 %)	78/94 (83.0 %)		0.690

TWSTRS, Toronto western spasmodic torticollis rating scale; CGI, clinical global impression; CGI-I, clinical global impression of illness; PGI, Patient's global impression; PGI-I, Patient's global impression of improvement.

a The proportions of patients with CGI of illness (CGI-I) of ‘1 = normal/not at all ill'or ‘2 = borderline mildly ill' or ‘3 = mildly ill' and PGI of improvement (PGI-I) of ‘1 = very much improved' or ‘2 = much improved' or ‘3 = mildly improved' were compared for each month follow-up.

No significant differences were seen in the proportion of CGI-I or PGI-I over the period containing all follow-up visits. In addition, no detectable differences in the TWSTRS subscale were found over the entire follow-up period (Table2).

Thirty-six patients preferred Dysport, and 34 chose Botox. Twenty-one patients said there was no difference between two treatments. In the group that preferred Dysport, the preference reasons were better efficacy (n = 25), longer duration of action (n = 8), decreased side effects (n = 9), and more tolerable pain (n = 5). The reasons for preferring Botox were better efficacy (n = 24), longer duration of action (n = 7), decreased side effects (n = 5), and more tolerable pain (n = 2).

### Safety Analysis

Adverse events were observed in 25 patients (14 in Dysport, 19 in Botox), and eight patients exhibited side effects in both regimens. Muscle weakness and dysphagia were the two most common AEs in both treatments; however, both effects were transient and tolerable. The incidence of AE was not significantly different between treatments (*P* = 0.332; Table3).

**Table 3:** Number of patients with adverse events in the modified intent-to-treat population

	Dysport (n = 94)	Botox (n = 94)	P value
Subjects with at least one AE, n (%)	14 (14.9)	19 (20.2)	0.332
AEs by category, n (%)			
Severe	0 (0.0)	0 (0.0)	1.000
Moderate	0 (0.0)	1 (1.1)	1.000
Treatment-related	14 (14.9)	19 (20.2)	0.332
Leading to discontinuation	0 (0.0)	0 (0.0)	1.000
AEs by type, n (%)			
Neck muscle weakness	9 (9.6)	13 (13.8)	0.388
Dysphagia	6 (6.4)	12 (12.8)	0.070
Pain on neck and shoulder	2 (2.1)	7 (7.4)	0.180
Local pain	2 (2.1)	1 (1.1)	1.000
Neck rigidity	1 (1.1)	1 (1.1)	1.000
Hoarseness	1 (1.1)	1 (1.1)	1.000
Headache	1 (1.1)	0 (0.0)	1.000
Dyspnea	0 (0.0)	1 (1.1)	1.000
Paresthesia	0 (0.0)	1 (1.1)	1.000
Dysarthria	0 (0.0)	1 (1.1)	1.000
Fatigue	0 (0.0)	1 (1.1)	1.000

## Discussion

Dysport, a commonly available preparation of BoNT-A, has been used in the treatment of CD patients, and its efficacy and safety has been demonstrated in multiple randomized, placebo-controlled studies.^16^–^18^ Moreover, treatment with 500 units Dysport is reported to be effective and safe in the treatment of CD when provided as initial or maintenance doses.^15^,^17^,^18^

A review suggests that the different formulations of BoNT-A are clinically similar and differ only in AEs.^19^ However, other literatures have reported intrinsic differences between formulations that render them non-bioequivalent, irrespective of dose ratios,^20^ and the conversion ratio may not be applicable.^21^

The use of BoNT-A in CD should be based on their individual dosing. However, when changing one preparation to the other because of any reasons, including economical or regional causes, we need minimum standards to avoid an overdose or an inefficient response.

A previous double-blinded study reported that Dysport treatment at a ratio of 3:1 was more effective than Botox in CD, but the incidence of AEs was somewhat higher with the 3:1 Dysport treatment.^8^ Therefore, use of a ratio lower than 3:1 was proposed. However, data for ratios lower than 3:1 were lacking, and a Dysport/Botox conversion ratio has been a matter of debate.^3^,^10^

To our knowledge, this is the first, double-blind randomized trial to compare Dysport and Botox at a ratio of 2.5:1 in the treatment of CD. We detected two significant results in the treatment of CD with Dysport and Botox. First, our study shows that Dysport treatment results were not inferior to those from Botox at a conversion factor of 2.5:1. Second, at the ratio of 2.5:1, the adverse event profile was similar in the treatment of CD. When using the 2.5:1 ratio in clinical practice, one vial of Dysport (500 units) corresponds to two vials of Botox (200 units).

The results in this study are consistent with those in articles that have proposed that an optimal Dysport/Botox ratio is lower than 3:1.^3^,^9^,^10^ In contrast to studies that used a 3:1 ratio, we detected no differences in efficacy or frequency of AE, suggesting that the 2.5:1 ratio is more appropriate for the treatment of CD. This ratio is supported by the results of hemifacial spasm,^22^ dematologic,^23^,^24^ and animal-based studies.^25^

The same volume was to be injected in each patient and in the same muscles in both study phases. However, injection sites in eight patients were different between the two phases, and those cases are reported as violating study protocols. In addition, one patient did not meet our inclusion criteria because of a short-duration disease. Thus, nine patients were excluded from the PP analysis, leaving 85 patients (82.5%) included in that analysis. The PP analysis showed no significant differences between Dysport and Botox when used at a 2.5:1 ratio (Supplemental Data Table e-2).

Our study used doses lower than 500 units Dysport and 200 units Botox. This finding is consistent with an article that suggested that less than 500 units Dysport was recommended as optimal dose in CD, considering dose-related response and AE.^16^ In other Botox studies, a clear dose-related response was not detected; however, 100 to 150 units was recommended.^26^ Even though our dose range is lower than the usual dose, a recent double-blind, randomized, placebo-controlled multicenter trial by Fernandez et al.^27^ showed that a lower starting dose (120 unit vs. 240 unit) may be better tolerated among toxin-naïve subjects without sacrificing efficacy for the other type of BoNT-A, Xeomin.^27^ Lower body weight and ethnicity also could be responsible for a lower dose of BoNT-A in our group. Almost all CD patients in Korea receive Dysport less than 500 units or Botox less than 200 units. Considering individual needs and ethnic difference, studies testing conversion ratios at higher doses for Dysport and Botox can be considered.

Dysphagia is most common AE observed with BoNT-A treatment in CD.^28^ In our results, the incidence of dysphagia was lower than in other randomized double-blind studies.^7^,^17^,^18^ Low dose in this study can be the explanation, because a meta-analysis by Chapman et al.^20^ showed that dysphagia is dose-dependent for Dysport. Additionally, Dysport treatment was associated with a significantly higher rate of dysphagia than Botox at ratios of 3:1 to 4:1,^8^ implying overdosing of Dysport at those ratios. These findings also imply that the ratio of 2.5:1 is more appropriate for the treatment of CD.

The interpretation of crossover studies can be confounded by carryover effects. To avoid carryover effects, we used a 4-week washout period between the 16-week interventions. No significant carryover effects were detected (Supplemental Data Table e-3). However, additional analysis on the data for the first intervention period only was done to eliminate the influence of potential carryover effects, and the results were not significantly different (Supplemental Data Table e-4).

In conclusion, our results suggest that Dysport and Botox when applied at a 2.5:1 ratio are similarly effective and well tolerated for the treatment of CD. These results could have clinical implications in the treatment of other neurological disorders; however, more clinical trials are needed to apply this ratio to other indications or clinical practices that require higher doses. Because the commonly available medication vials contain 500 units of Dysport or 100 units of Botox, the use of a 2.5:1 ratio is relatively easy to accomplish in clinical practice. This study is also supportive of the idea that lower than 500 units Dysport is optimal in CD.

## References

[1] Chan J, Brin MF, Fahn S (1991). Idiopathic cervical dystonia: clinical characteristics. Mov Disord.

[2] Simpson DM, Blitzer A, Brashear A (2008). Assessment: botulinum neurotoxin for the treatment of movement disorders (an evidence-based review): report of the Therapeutics and Technology Assessment Subcommittee of the American Academy of Neurology. Neurology.

[3] Ravenni R, De Grandis D, Mazza A (2013). Conversion ratio between Dysport and Botox in clinical practice: an overview of available evidence. Neurol Sci.

[4] Rystedt A, Nyholm D, Naver H (2012). Clinical experience of dose conversion ratios between 2 botulinum toxin products in the treatment of cervical dystonia. Clin Neuropharmacol.

[5] Brockmann K, Schweitzer K, Beck G, Wachter T (2012). Comparison of different preparations of botulinumtoxin A in the treatment of cervical dystonia. Neurology Asia.

[6] Bihari K (2005). Safety, effectiveness, and duration of effect of BOTOX after switching from Dysport for blepharospasm, cervical dystonia, and hemifacial spasm dystonia, and hemifacial spasm. Curr Med Res Opin.

[7] Odergren T, Hjaltason H, Kaakkola S (1998). A double blind, randomised, parallel group study to investigate the dose equivalence of Dysport and Botox in the treatment of cervical dystonia. J Neurol Neurosurg Psychiatry.

[8] Ranoux D, Gury C, Fondarai J, Mas JL, Zuber M (2002). Respective potencies of Botox and Dysport: a double blind, randomised, crossover study in cervical dystonia. J Neurol Neurosurg Psychiatry.

[9] Poewe W (2002). Respective potencies of Botox and Dysport: a double blind, randomised, crossover study in cervical dystonia. J Neurol Neurosurg Psychiatry.

[10] Wohlfarth K, Sycha T, Ranoux D, Naver H, Caird D (2009). Dose equivalence of two commercial preparations of botulinum neurotoxin type A: time for a reassessment?. Curr Med Res Opin.

[11] Jankovic J (2004). Treatment of cervical dystonia with botulinum toxin. Mov Disord.

[12] Tarsy D (1997). Comparison of clinical rating scales in treatment of cervical dystonia with botulinum toxin. Mov Disord.

[13] Consky E, Basinski A, Belle L, Ranawaya R, Lang A (1990). The Toronto Western Spasmodic Torticollis Rating Scale (TWSTRS): assessment of validity and inter-rater reliability. Neurology.

[14] Comella CL, Jankovic J, Shannon KM (2005). Comparison of botulinum toxin serotypes A and B for the treatment of cervical dystonia. Neurology.

[15] Truong D, Brodsky M, Lew M (2010). Long-term efficacy and safety of botulinum toxin type A (Dysport) in cervical dystonia. Parkinsonism Rel Disord.

[16] Poewe W, Deuschl G, Nebe A (1998). What is the optimal dose of botulinum toxin A in the treatment of cervical dystonia? Results of a double blind, placebo controlled, dose ranging study using Dysport. German Dystonia Study Group. J Neurol Neurosurgery Psychiatry.

[17] Wissel J, Kanovsky P, Ruzicka E (2001). Efficacy and safety of a standardised 500 unit dose of Dysport (clostridium botulinum toxin type A haemaglutinin complex) in a heterogeneous cervical dystonia population: results of a prospective, multicentre, randomised, double-blind, placebo-controlled, parallel group study. J Neurol.

[18] Truong D, Duane DD, Jankovic J (2005). Efficacy and safety of botulinum type A toxin (Dysport) in cervical dystonia: results of the first US randomized, double-blind, placebo-controlled study. Mov Disord.

[19] Sampaio C, Costa J, Ferreira JJ (2004). Clinical comparability of marketed formulations of botulinum toxin. Mov Disord.

[20] Chapman MA, Barron R, Tanis DC, Gill CE, Charles PD (2007). Comparison of botulinum neurotoxin preparations for the treatment of cervical dystonia. Clin Ther.

[21] Marchetti A, Magar R, Findley L (2005). Retrospective evaluation of the dose of Dysport and BOTOX in the management of cervical dystonia and blepharospasm: the REAL DOSE study. Mov Disord.

[22] Kollewe K, Mohammadi B, Dengler R, Dressler D (2010). Hemifacial spasm and reinnervation synkinesias: long-term treatment with either Botox or Dysport. J Neural Transm.

[23] Lowe P, Patnaik R, Lowe N (2006). Comparison of two formulations of botulinum toxin type A for the treatment of glabellar lines: a double-blind, randomized study. J Am Acad Dermatol.

[24] Hexsel D, Dal'Forno T, Hexsel C, Do Prado DZ, Lima MM (2008). A randomized pilot study comparing the action halos of two commercial preparations of botulinum toxin type A. Dermatol Surg.

[25] Rosales R, Bigalke H, Dressler D (2006). Pharmacology of botulinum toxin: differences between type A preparations. Eur J Neurol.

[26] Gelb DJ, Lowenstein DH, Aminoff MJ (1989). Controlled trial of botulinum toxin injections in the treatment of spasmodic torticollis. Neurology.

[27] Fernandez HH, Pappert EJ, Comella CL (2013). Efficacy and safety of incobotulinumtoxina in subjects previously treated with botulinum toxin versus toxin-naive subjects with cervical dystonia. Tremor Other Hyperkinet Mov (N Y).

[28] Borodic GE, Joseph M, Fay L, Cozzolino D, Ferrante RJ (1990). Botulinum A toxin for the treatment of spasmodic torticollis: dysphagia and regional toxin spread. Head Neck.

