# Supplementary material for: Dysport and Botox at a Ratio of 2.5:1 Units in Cervical Dystonia: A Double-Blind, Randomized Study
Source: Mov Disord. 2014 Dec 5;30(2):206–13. doi: 10.1002/mds.26085 (PMC4359015; doi:10.1002/mds.26085)
Supplement: Supplementary file 2 [file mds0030-0206-sd2.docx]

**Table e-1.** Clinical variables in each visit in the modified intention-to-treat population.

| **Scale** | **Time point** | **Dysport^®^  (N = 94)** | **Botox^®^ (N = 94)** | **Between-treatment difference in end-point** | **P value** |
| --- | --- | --- | --- | --- | --- |
| **Mean changes of Total Tsui from baseline** | 4 weeks | -3.98 ± 3.89 | -4.77 ± 4.10 | 0.78 [-0.13 to 1.70] | 0.091 |
|  | 8 weeks | -4.33 ± 3.69 | -4.23 ± 4.08 | -0.10 [-1.01 to 0.82] | 0.836 |
|  | 12 weeks | -3.20 ± 4.48 | -3.31 ± 4.18 | 0.11 [-0.99 to 1.21] | 0.848 |
|  | 16 weeks | -1.69 ± 3.99 | -1.85 ± 4.16 | 0.15 [-0.94 to 1.26] | 0.775 |
| **Mean changes of Total TWSTRS from baseline** | 4 weeks | -9.76 ± 10.25 | -8.78 ± 10.11 | -0.97 [-3.39 to 1.45] | 0.429 |
|  | 8 weeks | -11.16 ± 10.85 | -9.98 ± 9.90 | -1.18 [-3.58 to 1.23] | 0.333 |
|  | 12 weeks | -8.95 ± 11.64 | -8.66 ± 10.45 | -0.29 [-3.05 to 2.47] | 0.837 |
|  | 16 weeks | -5.15 ± 8.73 | -5.34 ± 9.76 | 0.18 [-2.43 to 2.80] | 0.889 |
| **Mean changes of TWSTRS severity subscore** | 4 weeks | -5.55 ± 4.99 | -5.26 ± 4.79 | -0.30 [-1.46 to 0.86] | 0.611 |
|  | 8 weeks | -5.95 ± 5.13 | -5.12 ± 4.80 | -0.83 [-1.99 to 0.33] | 0.159 |
|  | 12 weeks | -4.61 ± 5.50 | -4.11 ± 5.08 | -0.67 [-1.82 to 0.82] | 0.456 |
|  | 16 weeks | -2.41 ± 4.45 | -1.85 ± 4.75 | -0.64 [-1.84 to 0.71] | 0.381 |
| **Mean changes of TWSTRS disability subscore** | 4 weeks | -2.76 ± 3.64 | -2.46 ± 3.60 | -0.30 [-1.23 to 0.64] | 0.529 |
|  | 8 weeks | -3.24 ± 4.17 | -2.70 ± 3.80 | -0.54 [-1.52 to 0.43] | 0.272 |
|  | 12 weeks | -2.94 ± 4.52 | -2.61 ± 3.84 | -0.33 [-1.46 to 0.80] | 0.564 |
|  | 16 weeks | -1.74 ± 3.63 | -1.84 ± 3.74 | 0.10 [-0.95 to 1.14] | 0.856 |
| **Mean changes of TWSTRS pain subscore** | 4 weeks | -1.45 ± 4.05 | -1.19 ± 4.16 | -0.25 [-1.28 to 0.77] | 0.623 |
|  | 8 weeks | -1.96 ± 3.97 | -2.28 ± 3.50 | 0.32 [-0.62 to 1.26] | 0.498 |
|  | 12 weeks | -1.40 ± 4.08 | -2.07 ± 3.97 | 0.67 [-0.44 to 1.78] | 0.234 |
|  | 16 weeks | -0.97 ± 3.85 | -1.76 ± 3.75 | 0.79 [-0.37 to 1.95] | 0.180 |
| **Number of patients (%) scoring 1 or 2 or 3 on CGI scale (CGI-I)†** | Baseline | 25/94 (26.6 %) | 24/94 (25.5 %) |  | 1.000 |
|  | 4 weeks | 54/94 (57.4 %) | 57/94 (60.6%) |  | 0.690 |
|  | 8 weeks | 66/94 (70.2 %) | 58/94 (61.7 %) |  | 0.115 |
|  | 12 weeks | 57/94 (60.6 %) | 57/94 (60.6 %) |  | 1.000 |
|  | 16 weeks | 43/94 (45.7 %) | 41/94 (43.6 %) |  | 0.855 |
| **Number of patients (%) scoring 1 or 2 or 3 on PGI scale (PGI-I)†** | 4 weeks | 75/94 (79.8 %) | 78/94 (83.0 %) |  | 0.648 |
|  | 8 weeks | 77/94 (81.9 %) | 78/94 (83.0 %) |  | 1.000 |
|  | 12 weeks | 72/94 (76.6 %) | 72/94 (76.6 %) |  | 1.000 |
|  | 16 weeks | 51/94 (54.3 %) | 50/94 (53.2 %) |  | 1.000 |

TWSTRS, Toronto western spasmodic torticollis rating scale; CGI, clinical global impression; CGI-I, clinical global impression of illness; PGI, Patient’s global impression; PGI-I, Patient’s global impression of improvement.

†The proportions of patients with CGI of illness (CGI-I) of ‘1 = normal/not at all ill’or ‘2 = borderline mildly ill’ or ‘3 = mildly ill’ and PGI of improvement (PGI-I) of ‘1 = very much improved’ or ‘2 = much improved’ or ‘3 = mildly improved’ were compared for each month follow-up.
