# Supplementary material for: Dysport and Botox at a Ratio of 2.5:1 Units in Cervical Dystonia: A Double-Blind, Randomized Study
Source: Mov Disord. 2014 Dec 5;30(2):206–13. doi: 10.1002/mds.26085 (PMC4359015; doi:10.1002/mds.26085)
Supplement: Supplementary file 3 [file mds0030-0206-sd3.docx]

**Table e-2.** Clinical outcomes after 4 weeks from baseline in the per-protocol population

| **Scale** | **Dysport^®^  (N = 85)** | **Botox^®^ (N = 85)** | **Between-treatment difference in end-point** | **P value** |
| --- | --- | --- | --- | --- |
| **Mean changes of Total Tsui from baseline** | -3.62 ± 3.76 | -4.42 ± 4.05 | 0.80 [-0.20 to 1.80] | 0.114 |
| **Mean changes of Total TWSTRS from baseline** | -9.13 ± 10.03 | -7.80 ± 9.93 | -1.32 [-3.39 to 1.45] | 0.300 |
| **Mean changes of TWSTRS severity subscore** | -5.18 ± 4.88 | -4.81 ± 4.69 | -0.36 [-1.60 to 0.87] | 0.558 |
| **Mean changes of TWSTRS disability subscore** | -2.55 ± 3.56 | -2.08 ± 3.48 | -0.47 [-1.43 to 0.48] | 0.330 |
| **Mean changes of TWSTRS pain subscore** | -1.40 ± 4.09 | -1.01 ± 4.24 | -0.39 [-1.74 to 0.22] | 0.487 |
| **Number of patients (%) scoring 1 or 2 or 3 on CGI scale (CGI-I)†** | 48/85 (56.5 %) | 50/85 (58.8 %) |  | 0.839 |
| **Number of patients (%) scoring 1 or 2 or 3 on PGI scale (PGI-I)†** | 66/85 (77.6 %) | 70/85 (82.4 %) |  | 0.481 |

TWSTRS, Toronto western spasmodic torticollis rating scale; CGI, clinical global impression; CGI-I, clinical global impression of illness; PGI, Patient’s global impression; PGI-I, Patient’s global impression of improvement.

†The proportions of patients with CGI of illness (CGI-I) of ‘1 = normal/not at all ill’or ‘2 = borderline mildly ill’ or ‘3 = mildly ill’ and PGI of improvement (PGI-I) of ‘1 = very much improved’ or ‘2 = much improved’ or ‘3 = mildly improved’ were compared for each month follow-up.
