# Supplementary material for: Dysport and Botox at a Ratio of 2.5:1 Units in Cervical Dystonia: A Double-Blind, Randomized Study
Source: Mov Disord. 2014 Dec 5;30(2):206–13. doi: 10.1002/mds.26085 (PMC4359015; doi:10.1002/mds.26085)
Supplement: Supplementary file 4 [file mds0030-0206-sd4.docx]

**Table e-3.** Carryover effects.

|  | **Arm 1 (Dysport^®^ → Botox^®^)** | **Arm 2 (Botox^®^ → Dysport^®^)** | **P value** |
| --- | --- | --- | --- |
| **Mean change of total Tsui at 4 weeks** | -0.78 ± 3.31 | -1.19 ± 3.68 | 0.915 |
| **Mean change of total TWSTRS at 4 weeks** | -5.07 ± 8.01 | -3.85 ± 9.52 | 0.501 |
| **Mean change of total TWSTRS severity subscore at 4 weeks** | -1.80 ± 3.38 | -0.32 ± 4.86 | 0.092 |
| **Mean change of total TWSTRS disability subscore at 4 weeks** | -1.74 ± 3.86 | -1.40 ± 3.95 | 0.717 |
| **Mean change of total TWSTRS pain subscore at 4 weeks** | -1.27 ± 3.48 | -2.20 ± 4.17 | 0.277 |

Carryover calculated as (Visit6-Visit1)Arm1 - (Visit6-Visit1)Arm2.

Mann-Whitney U test.
