# Supplementary material for: Dysport and Botox at a Ratio of 2.5:1 Units in Cervical Dystonia: A Double-Blind, Randomized Study
Source: Mov Disord. 2014 Dec 5;30(2):206–13. doi: 10.1002/mds.26085 (PMC4359015; doi:10.1002/mds.26085)
Supplement: Supplementary file 5 [file mds0030-0206-sd5.docx]

**Table e-4.** Clinical variables in the first session.

| **Scale** | **Time point** | **Dysport^®^ (N = 46)** | **Botox^®^ (N = 48)** | ***P* value** |
| --- | --- | --- | --- | --- |
| **Mean changes of Total Tsui from baseline** | 4 weeks | -5.14 ± 3.94 | -5.27 ± 3.71 | 0.690 |
|  | 8 weeks | -5.60 ± 4.07 | -4.60 ± 3.75 | 0.282 |
|  | 12 weeks | -4.83 ± 4.87 | -4.21 ± 3.84 | 0.554 |
|  | 16 weeks | -2.63 ± 4.42 | -2.54 ± 4.27 | 0.939 |
| **Mean changes of Total TWSTRS from baseline** | 4 weeks | -13.28 ± 11.64 | -10.93 ± 10.36 | 0.436 |
|  | 8 weeks | -14.84 ± 12.35 | -12.23 ± 9.88 | 0.307 |
|  | 12 weeks | -13.36 ± 13.18 | -11.38 ± 10.64 | 0.771 |
|  | 16 weeks | -8.17 ± 9.59 | -7.53 ± 10.35 | 0.745 |
| **Mean changes of TWSTRS severity subscore** | 4 weeks | -6.89 ± 5.26 | -6.17 ± 4.67 | 0.434 |
|  | 8 weeks | -7.74 ± 5.11 | -5.77 ± 4.75 | 0.054 |
|  | 12 weeks | -6.61 ± 5.24 | -5.42 ± 4.60 | 0.446 |
|  | 16 weeks | -3.89 ± 4.28 | -2.65 ± 4.91 | 0.221 |
| **Mean changes of TWSTRS disability subscore** | 4 weeks | -3.98 ± 3.95 | -3.06 ± 3.93 | 0.262 |
|  | 8 weeks | -4.59 ± 4.66 | -3.06 ± 4.33 | 0.347 |
|  | 12 weeks | -4.59 ± 4.66 | -3.46 ± 4.28 | 0.244 |
|  | 16 weeks | -2.59 ± 3.98 | -2.21 ± 4.34 | 0.374 |
| **Mean changes of TWSTRS pain subscore** | 4 weeks | -2.41 ± 4.83 | -1.68 ± 4.42 | 0.590 |
|  | 8 weeks | -2.51 ± 4.70 | -3.00 ± 3.52 | 0.533 |
|  | 12 weeks | -2.19 ± 4.82 | -2.90 ± 4.10 | 0.209 |
|  | 16 weeks | -1.67 ± 4.13 | -2.67 ± 3.82 | 0.143 |

TWSTRS, Toronto western spasmodic torticollis rating scale.

Mann-Whitney U test.
